# Differences in Physical Activity Levels, Screen Time, and Body Mass Index and Their Associations with Oral Health in Schoolchildren in Mallorca

**DOI:** 10.3390/children11111280

**Published:** 2024-10-24

**Authors:** Irene Coll, Daniela Vallejos, Nora López-Safont

**Affiliations:** 1 Facultad of Dentistry, University ADEMA School, 07009 Palma, Spain; i.coll@eua.edu.es (I.C.); d.vallejos@eua.edu.es (D.V.); 2 Health Group of University Institute for Research in Health Sciences (IUNICS), ADEMA, 07009 Palma, Spain; 3 Biology Department, University of Balearic Islands, Calle Passamaners 11, 07009 Palma, Spain

**Keywords:** body mass index, oral health, screen time, sex, physical activity

## Abstract

Background: The time that adolescents spend using screens is associated with an elevated body mass index (BMI) and decreased physical activity, with gender being an important determinant. There is evidence that an elevated BMI can affect oral health. Aim: To analyze gender differences in physical activity levels and screen time, as well as screen use and BMI, and their associations with DMFT. Methods: Physical activity levels, screen time, and their relationship with the oral health status and BMI were studied in 468 schoolchildren aged 12 to 15 years. To analyze the differences in the numerical data, an analysis of the mean by the Student *t*-test or a one-way analysis of variance followed by the Bonferroni post hoc analysis was used. Moreover, to analyze the differences in the categorical data, the chi-square test was used. Results: Boys were more active (59.9%) than girls (40.1%) (*p* < 0.001) in terms of the means of transportation that they used to travel to school. It was observed that boys spent more mean hours playing outdoors than girls (boys: 1.38 ± 0.04 vs. girls: 1.24 ± 0.04; *p* = 0.040). The mean number of hours engaged in sports activities outside school was higher for boys than girls (boys: 2.22 ± 0.06 vs. girls: 1.77 ± 0.73; *p* ≤ 0.001). The mean number of hours spent using electronic devices during the weekend was higher in boys than girls (boys: 2.89 ± 0.08 vs. girls 2.44 ± 0.09; *p* ≤ 0.001). Children with a DMF > 0 had a higher mean BMI than those with a DMFT = 0 (DMFT > 0; 21.95 ±4.80 vs. DMFT = 0; 20.77 ± 3.67; *p* = 0.003). Conclusions: An increased number of hours spent in front of a computer correlates with a higher BMI. Sex seems to be a determining factor when it comes to engaging in active activities. Caries is more frequent in children with a higher BMI.

## 1. Introduction

Health determinants, including lifestyles and living conditions, play an important role in an individual’s development [1].

The adolescent years are particularly critical for the establishment of lifestyles, during which the reinforcement of certain childhood habits occurs, with the adoption of new ones learned through socialization scenarios [2].

This behavior is conditioned by childhood experiences. Children can accumulate physical activity throughout the day through different behaviors, such as active transportation (e.g., walking or biking) to school, participation in sports or organized activities, and playing outdoors or indoors [3]. However, it is estimated that, globally, the prevalence of sufficient physical activity in schoolchildren and adolescents is only 19.3% [4].

One element contributing to an unhealthy lifestyle is the rise in passive or motorized transportation for many daily excursions, particularly from home to school [5].

There is growing awareness that extracurricular activities are an important resource to stimulate optimal adolescent development [2]. Generally, the preferred activities in adolescence include socializing with friends, followed by watching television, listening to music, playing sports, going to the movies, reading books, sleeping/resting, doing nothing, and traveling [6]. In other words, there is little participation in structured activities [6]. Regarding gender, it is noted that girls exhibit markedly lower engagement in extracurricular activities than boys, likely due to the predominance of sports, particularly soccer, among these activities [6].

Results found in the Health Behaviour in School-Aged Children (HBSC) project, funded by the WHO since 1982 and involving 25 countries, show that boys are more physically active than girls in every European country, with Spain showing the greatest gender differences [7]. Generally, girls participate in sports less frequently, less intensely, and for shorter durations than boys [7].

The decline in regular physical exercise has been intensified, among other factors, by the rise in technological leisure activities that encourage a sedentary lifestyle [8]. In recent years, the amount of screen time has been increasing [9], gaining ground among the routines of Spaniards, especially the youngest [9,10].

Previous studies in the United States show that, among adolescents aged 12 to 19 years, 83% use a smart device [11].

There is evidence that increased screen time correlates with obesity [12,13,14], which is believed to occur through several mechanisms, such as increased caloric intake, decreased resting metabolism, a reduced sleep duration, and a lack of physical activity due to a shift in the time spent on it [15]. The WHO does not recommend that children and adolescents be exposed to these devices for more than two hours per day [16]. Regarding gender differences, several studies conclude that boys spend more time playing video games than girls [17,18].

The ALADINO study reveals that overweight in Spanish children has stabilized in the last ten years, affecting 45.2% of children between the ages of 6 and 9; this represents a major public health issue. Of these 45.2%, 26.1% are overweight, and 19.1% are obese. Regarding gender, boys are more overweight than girls (26.3% overweight vs. 25.9% and 22% obese vs. 16.2%) [19].

Obesity is considered an important risk factor for non-communicable diseases, such as cardiovascular diseases, type 2 diabetes mellitus, musculoskeletal disorders, and some cancers (endometrium, breast, and colon) [20,21].

Several studies relate screen use with obesity and oral health [22,23,24]. A longer television viewing time is significantly and verifiably associated with a larger number of decayed teeth and a higher DMF (“decayed, missing, and filled”) index [24].

Moreover, many oral health behaviors significantly affect physical and psychosocial health during adolescence, while others have potentially negative effects on one’s future health status, as oral hygiene behaviors show high continuity between adolescence and adulthood [25].

Oral health significantly influences physical well-being and social dynamics, particularly pain and functionality, as measured by dietary constraints, communication barriers, discomfort, and aesthetic dissatisfaction [26]. Caries and obesity are diseases with similar risk factors, such as diet, genetic and non-genetic factors, socioeconomic status, environmental factors, and lifestyle [27].

Studies have sought to link weight and caries because most health problems associated with growth, development, and oral diseases share a common pathway: diet. Although some have found a relationship, the results have been mixed or contradictory [28]. The results of several studies demonstrate the link between dental caries and the body mass index (BMI), with the mean number of dental caries being higher in overweight and obese schoolchildren [29,30].

It is common for studies on child/adolescent health to focus on individual topics such as obesity, physical activity, or oral health, but they do not always integrate these aspects into a single analysis. This study aimed to analyze the gender differences in physical activity levels and screen time, as well as between screen use and the BMI. The analysis also included the examination of the caries history (DMFT levels) and its correlation with students’ BMI.

## 2. Materials and Methods

### 2.1. Study Design and Target Population

This observational epidemiological study was designed according to the World Health Organization (WHO) guidelines for conducting oral health surveys using the Pathfinder method [31] and following the description in the ENALIA study to obtain data on physical activity and screen use [23,32]. The sample size was calculated in the following way: for a population of 12,000 children and a caries prevalence proportion of 0.35, to achieve a 95% confidence level with a 5% margin of error, the minimum sample size was approximately 340 children.

The school population was the target of the present study, with 2 cohorts of index ages recommended by the WHO: 12 and 15 years [31]. A total of 463 adolescents (12–15 years) were analyzed—specifically, 230 adolescents aged 12 years (girls *n* = 105, boys *n* = 125) and 233 adolescents aged 15 years (girls *n* = 121, boys *n* = 112), maintaining gender equity. The strata included were the population center (urban, peri-urban, and rural centers), type of school (public and charter/private), and age group (12 and 15 years). After segmenting the population into various strata, systematic random sampling was used to select the schools, ensuring the representativeness of each stratum by applying the proportionality criterion according to the characteristics of the study area.

The present study was approved by the Research Ethics Committee of the Balearic Islands (CEI: IB3737/18, 17 September 2018) in accordance with the current legislation and was conducted in line with the principles contained in the Declaration of Helsinki and the standards of good clinical practice. Before starting the study, information was provided to the students’ parents or guardians (they received the study information sheet and the informed consent form), and only those children whose parents or guardians signed this were included in the study.

### 2.2. Data Collection and Study Variables

Oral health (DMFT), physical condition (BMI), sex, hours of screen time, and hours of physical activity variables were recorded between November 2018 and December 2019. The DMFT data collection sheet was extracted from “Oral health surveys: basic methods” [30], using standardized lighting conditions (head light), instruments (dental mouth mirror #5 and WHO periodontal probe), and examinee positions. The data collection sheet on screen time and hours of physical activity was extracted from a physical activity questionnaire based on the International Physical Activity Questionnaire (IPAQ), validated and used in Spanish studies like the ENALIA study [33]. The subjects’ anthropometric measurements, consisting of weight and height, were also recorded.

The variables considered for the analysis were sex, DMFT (sum of decayed, missing, and filled permanent teeth), DMFT = 0 (no history of caries), DMFT > 0 (history of caries), weight, BMI (body mass index = weight (kg)/height^2^), hours of screen time (weekend computer/console hours; weekday television hours), and hours of physical activity (means of transportation to school).

### 2.3. Statistical Analysis

The data were analyzed using the SPSS 27.0.1.0^®^ statistics application. Numerical variables were expressed as means ± standard deviation, while nominal variables were expressed as percentages. Depending on the type of variable and the groups to be analyzed, differences were determined using Student’s *t*-test to examine the mean or by one-way analysis of variance (ANOVA) followed by Bonferroni post hoc analysis. Pearson’s bivariate correlation analysis was used for correlation analysis. The chi-square test was used to compare categorical variables. To obtain a measure of the precision (of the random error present in the data), the 95% confidence interval estimate (*p* < 0.05) was used in every case.

## 3. Results

### 3.1. Differences in Means of Transportation Used to Travel to School and Students’ Sex

Sex conditions the means of transportation used by students to travel to school; it is observed that boys are more active (59.9%) than girls (40.1%) (*p* < 0.001) when it comes to traveling to school. The use of bicycles (as an active element) is exhibited by 78.1% of boys, compared to only 21.9% of girls. On the other hand, the bus (as a passive element) is used by 57.4% of girls, compared to 42.6% of boys (Table 1).

### 3.2. Differences in Hours of Outdoor Activity and Students’ Sex

Sex also influences the students’ hours of activity; boys spend an average of 50.77% more hours playing outdoors during the week than girls (boys: 1.38 ± 0.04 vs. girls: 1.24 ± 0.04; *p* = 0.040). Moreover, the mean number of hours engaged in sports/dance activities outside school hours is 53.38% higher for boys than girls (boys: 2.22 ± 0.06 vs. girls: 1.77 ± 0.73; *p* ≤ 0.001) (Table 2).

### 3.3. Differences in Hours of Screen Time According to Students’ BMI

We noted a tendency for adolescents with a higher BMI to spend more hours in front of screens on weekdays (none: 20.33 ± 0.38; less than 1 h or about 1 h: 21.35 ± 0.32; 2 h or more: 21.62 ± 0.31; *p* = 0.05) (Table 3). Students who exhibit at least 1 h of screen time during the week tend to have a higher BMI than those who do not use screens. Overall, 78.92% of the school population studied in Mallorca spends 1 h or more in front of a screen.

In this context, no significant differences were observed in terms of gender (see Table 4).

### 3.4. Differences in Screen Time and Sex

We observed significant differences concerning gender and the mean number of hours spent using computers, consoles, and similar devices on the weekend (boys: 2.89 ± 0.08 vs. girls 2.44 ± 0.09; *p* ≤ 0.001) (Table 5).

We observed that boys spent a larger mean number of hours in front of the television than girls during the week (boys: 1.84 ± 0.08 vs. girls 1.60 ± 0.8; *p* = 0.039) (Table 5).

Likewise, the same difference was observed on weekends, with boys being 13.15% more likely to spend more time in front of screens than girls during the week and 15.54% more likely to spend more time in front of screens on weekends (*p* ≤ 0.001).

### 3.5. Differences in DMFT Level and BMI

**Figure 1:**
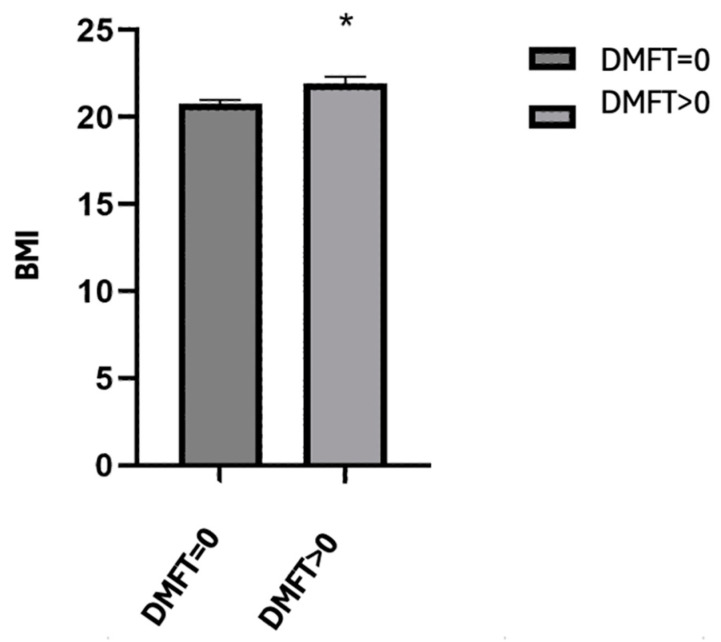
Mean BMI according to DMF index. The results represent the mean ± SD with * significant differences (Student’s *t*-test, *p* < 0.05).

In relation to the students’ physical condition and its influence on oral health, we noted that children with a DMFT > 0 (i.e., history of caries) had a higher mean BMI than those with a DMFT = 0 (DMFT > 0; 21.95 ± 4.80  vs. DMFT = 0; 20.77 ± 3.67; *p* = 0.003) (see Table 6 and Figure 1).

## 4. Discussion

The relationship between physical activity and health has been fully demonstrated by numerous scientific studies that, among other conclusions, reveal the positive impact of physical exercise in preventing obesity and the negative effects of sedentary lifestyles on health [34].

The recommendation for school-aged children is to dedicate at least 60 min daily to moderate to vigorous physical activity throughout the week and limit time engaged in sedentary activities. These recommendations pertain to the beneficial effects on the physical, mental, and social well-being of this demographic, where this life stage is critical for the establishment of healthy living practices [32,35,36].

Among the factors that explain a physically unhealthy lifestyle is the increase in the use of passive or motorized transport for the different journeys that a child makes during the day, mainly from home to school [5,35]. As in our study, several authors confirm that boys use more active transport than girls, who use the car more to travel to school [37,38]. Other authors reveal no differences between boys and girls in terms of active or motorized travel [39].

According to data from the PASOS 2022 study, children and adolescents aged 8 to 16 spend 6.7 min less on daily physical activity than in 2019, a concerning reduction in only 3 years [40].

According to the literature, sex seems to be a differentiating factor in the number of hours of physical activity undertaken by schoolchildren [41,42]. Several studies [42,43] exhibit the same findings as in our study, where boys dedicate more hours to physical activity both during the week and on weekends than girls. The decline in the time spent by girls in physical activity is concerning [40].

Reimers et al. identified that the time spent by girls engaging in physical activity in parks was affected by the number of people and the number of opposite-sex peers, factors that also carry over to the school environment [44].

International evidence illustrates that children and adolescents exhibit reduced levels of physical activity, favoring technological entertainment that requires minimal energy expenditure [45]. This trend progressively detaches them from consistent physical activity, potentially resulting in significant repercussions for their physical and cognitive development in the short, medium, and long term [45], including weight gain, cardiometabolic issues, and psychological challenges related to self-esteem and social interactions [45].

The results of our study indicate that children who spend at least 1 h in front of a computer during the week have a higher BMI than those who do not use a computer. Likewise, those schoolchildren who spend 2 h or more using a computer at the weekend have a higher BMI than those who spend less than an hour in front of a screen. Consistent with our findings, earlier studies from the 1990s indicated a 19.6% weight gain in children who spent increased hours watching television, suggesting a potential link between television exposure and childhood obesity [46]. Another more recent study also concludes that obesity is associated with screen time [47]. Many young people do not comply with the recommendations for screen time (≤2 h/day) [48]. However, a 2019 Royal College of Paediatrics and Child Health report proposes that the evidence is relatively weak and that no specific or universal screen time limit can be recommended [49].

On the other hand, sex was also related to the number of hours of screen time. Our study found that boys spent more hours in front of a computer than girls. These results contrast those of similar studies, which indicate that girls spend more hours per day in front of screens [46,50].

In this context, we noted that university students’ BMI is conditioned by their childhood lifestyles (specifically, the hours of screen use and hours of physical activity), and this is one of the potential elements that can affect health in general and oral health in particular. In relation to oral health, we have observed that schoolchildren with a DMFT > 0 have a higher mean BMI than those with a DMFT = 0 (DMFT > 0; 21.95 ± 4.80  vs. DMFT = 0; 20.77 ± 3.67; *p* = 0.003).

It is known that dental caries and obesity are multifactorial diseases that affect most of the pediatric population [51]. Previous studies corroborate our findings, establishing a direct positive correlation between caries and the BMI [52,53], although some authors present contrary evidence [54,55].

Additional research is required to assess these variables using methods with enhanced validity and reliability, particularly for physical activity, by using accelerometers or pulsometers instead of questionnaires, which are less valid.

The study of the relationship between the BMI, screen time, physical activity levels, and oral health requires a multidisciplinary approach involving pediatrics, dentistry, nutrition, and public health, among other areas. In some cases, these disciplines do not collaborate sufficiently at the national or local level to generate joint research.

Factors such as increased screen time or a lack of physical activity in children and adolescents are relatively recent trends, and their impact on oral health may not have been a focus of research until recently. In some countries, child/youth health studies focus on problems of greater prevalence or concern, such as malnutrition or obesity, without necessarily associating these problems with oral health, especially in school [56,57].

The present study could be of great value to fill this gap, and it suggests an innovative approach in analyzing how lifestyle variables, such as physical activity and screen time, affect oral health and BMI in children.

One limitation of this work was that it was difficult to find studies (especially local or national) that addressed all of these factors together, as research in this area appears to be at an early stage or underexplored in many regions.

Furthermore, this study had the limitations of a cross-sectional study, as there was no follow-up, and this leads to limitations when it comes to establishing a causal relationship.

## 5. Conclusions

Our data indicate that an increased number of hours spent in front of a computer correlates with a higher BMI among the schoolchildren studied. Sex seems to be a determining factor when it comes to engaging in active activities during the week and outside school hours. Caries is more frequent in children with a higher BMI.

Further studies are needed to clarify the possible controversies found in the literature. Moreover, other variables, such as dietary and physical activity behaviors, could influence the studied variables.

It is essential to establish health promotion programs to inform parents and guardians about their children’s passive leisure activities and their potential health implications.

## Figures and Tables

**Table 1:** Means of transportation to school according to the students’ sex.

Sex	*n*	Bus/Trans	Bike	Walking	Other	*p* Value
Boys	231	42.6%	78.1%	55.2%	87.5%	<0.001 *
Girls	225	57.4%	21.9%	44.8%	12.5%	
Sex	*n*			Passive	Active	*p* value
Boys	231			42.6%	59.9%	<0.001 *
Girls	225			57.4%	40.1%	

(*n*, sample size; chi-square test, * variable with significant effect (*p* < 0.05).

**Table 2:** Mean hours of activity among students by sex.

	Sex	*n*	Mean	SD	SE	95% CI	*p* Value
Mean hours playing outdoors during the week	Boys	229	1.3886	0.70838	0.04681	(1.2964–1.4809)	0.040 *
	Girls	222	1.2477	0.74108	0.04974	(1.1497–1.3458)	
Mean hours of sports/dance activities outside school hours	Boys	197	2.2284	0.90554	0.06452	(2.1012–2.3557)	<0.001 *
	Girls	172	1.7733	0.96778	0.07379	(1.6276–1.9189)	

(*n*, sample size; SD, standard deviation; SE, standard error; CI, confidence interval; Student’s *t*-test, * variable with significant effect (*p* < 0.05)).

**Table 3:** BMI according to the mean hours spent using a computer, video game console, or similar during the week.

Hours of Screen Time	Number of Students	Mean BMI	SD	SE	CI	*p* Value
None	94	20.3381	3.72890	0.38461	(19.5743–21.1018)	0.050
<1 h or about 1 h	178	21.3524	4.31508	0.32343	(20.7141–21.9907)	
2 h or more	174	21.6238	4.19944	0.31836	(20.9954–22.2521)	

(*n*, sample size; SD, standard deviation; SE, standard error; CI, confidence interval; one-way ANOVA, *p* < 0.05, and Bonferroni post hoc analysis).

**Table 4:** BMI according to mean hours spent of computer, video game console, or similar during the week and sex.

Hours of Screen Time	Sex	Number of Students	Mean BMI	SD	SE	CI	*p* Value
None	Boys	56	20.5820	38.2065	0.51056	(19.5588–21.6051)	0.819
<1 h or about 1 h		78	21.3263	37.8905	0.42903	(20.4720–22.1806)	
2 h or more		93	21.3991	38.1058	0.39514	(20.6143–22.6143)	
None	Girls	38	19.987	36.0947	0.58553	(19.7923–21.1651)	
<1 h or about 1 h		100	21.3728	47.0.348	0.47035	(20.4395–22.3061)	
2 h or more		81	21.8817	46.1645	0.51294	(20.8609–22.9025)	

(*n*, sample size; SD, standard deviation; SE, standard error; CI, confidence interval; one-way ANOVA, *p* < 0.05 and Bonferroni post hoc analysis).

**Table 5:** Mean hours of screen time according to sex.

	Sex	*n*	Mean	SD	SE	95% CI	*p* Value
Computer/console hours—weekend	Boys	224	2.8929	1.22644	0.08195	(2.7193–3.0454)	<0.001 *
	Girls	212	2.4434	1.38427	0.09507	(2.2681–2.6419)	
Computer/console hours—weekdays	Boys	212	1.9245	1.48738	0.10215	(1.7232–2.1259)	0.877
	Girls	210	1.9333	1.36088	0.09391	(1.7482–2.1185)	
TV hours—weekend	Boys	212	2.2500	1.30937	0.08993	(2.0727–2.4273)	0.529
	Girls	210	2.1952	1.29592	0.08943	(2.0189–2.4715)	
TV hours—weekdays	Boys	231	1.84850	1.26095	0.08296	(1.6877–2.0227)	0.039 *
	Girls	223	1.6054	1.23623	0.08278	(1.4252–1.7597)	

(*n*, sample size; SD, standard deviation; SE, standard error; CI, confidence interval; Student’s *t*-test, * variable with significant effect (*p* < 0.05)).

**Table 6:** Mean BMI according to the DMF index.

		*n*	Mean	SD	SE	CI	*p* Value
BMI	DMFT = 0	293	20.7722	3.67164	0.21450	(20.3501–21.1944)	0.003 *
	DMFT > 0	166	21.9505	4.80716	0.37311	(21.2138–22.6872)	

(*n*, sample size; SD, standard deviation; SE, standard error; CI, confidence interval; Student’s *t*-test, * variable with significant effect (*p* < 0.05)).

## Data Availability

The datasets generated and/or analyzed during the current study are not publicly available as they are being utilized for ongoing purposes, but they are available from the corresponding author on reasonable request.
